# The role of the podiatrist in falls prevention

**DOI:** 10.1186/1757-1146-3-S1-P10

**Published:** 2010-12-20

**Authors:** Olga Frankowski

**Affiliations:** 1 University of Salford, Salford, UK

## Introduction

With 1 in 3 people aged 65 or over suffering a fall at least once a year, rising to 1 in 2 in those over 80yrs, falls alone are estimated to cost the NHS £4.3 million per day, amounting to £1.7 billion per year. The aim of this poster is to provide an overview of ‘The role of the podiatrist in falls prevention’.

## Methods

Critical literature review

## Results

With over 400 separate risk factors for falls described in the literature to include a history of falls, mobility impairment, muscle weakness, gait deficit, and polypharmacy, a multifactorial intervention approach is recommended. This said however, the role of the podiatrist in falls prevention is not always recognized or fully understood.

## Discussion

Based upon individual risk factors, podiatrists have an important role to in play in reducing the risk of falls by means of foot health care itself, patient education, health promotion, rehabilitation and mobility. This includes assessing and treating foot pain, identifying and correcting underlying biomechanical and gait abnormalities, prescribing exercise programmes and issuing foot health and footwear advice. Within falls prevention teams or services the role of the podiatrist can further involve undertaking the training of other clinical staff in the recognition of medical factors influencing postural stability, gait and footwear, and promoting the role of the podiatrist.

## Implications for practice

There is a need for podiatrists to actively market podiatry services with particular emphasis on their roles and responsibilities. Further strategies are also needed to enhance the role of the podiatrist in falls prevention in a bid to further improve podogeriatric care. This would aid to prevent unnecessary injury or trauma, improve quality of life, reduce the incidence of falls and lower morbidity and mortality rates as a direst result of a fall.

